# Modulation of fetoplacental growth, development and reproductive function by endocrine disrupters

**DOI:** 10.3389/fendo.2023.1215353

**Published:** 2023-10-03

**Authors:** Sanjay Basak, Saikanth Varma, Asim K. Duttaroy

**Affiliations:** ^1^ Molecular Biology Division, ICMR-National Institute of Nutrition, Indian Council of Medical Research, Hyderabad, India; ^2^ Department of Nutrition, Institute of Basic Medical Sciences, Faculty of Medicine, University of Oslo, Oslo, Norway

**Keywords:** in utero exposure, endocrine-disrupting chemicals, fetoplacental growth, bisphenol, glucocorticoids, leptin, bps

## Abstract

Maternal endocrine homeostasis is vital to a successful pregnancy, regulated by several hormones such as human chorionic gonadotropin, estrogen, leptin, glucocorticoid, insulin, prostaglandin, and others. Endocrine stress during pregnancy can modulate nutrient availability from mother to fetus, alter fetoplacental growth and reproductive functions. Endocrine disrupters such as bisphenols (BPs) and phthalates are exposed in our daily life's highest volume. Therefore, they are extensively scrutinized for their effects on metabolism, steroidogenesis, insulin signaling, and inflammation involving obesity, diabetes, and the reproductive system. BPs have their structural similarity to 17-β estradiol and their ability to bind as an agonist or antagonist to estrogen receptors to elicit an adverse response to the function of the endocrine and reproductive system. While adults can negate the adverse effects of these endocrine-disrupting chemicals (EDCs), fetuses do not equip themselves with enzymatic machinery to catabolize their conjugates. Therefore, EDC exposure makes the fetoplacental developmental window vulnerable to programming *in utero*. On the one hand prenatal BPs and phthalates exposure can impair the structure and function of the ovary and uterus, resulting in placental vascular defects, inappropriate placental expression of angiogenic growth factors due to altered hypothalamic response, expression of nutrient transporters, and epigenetic changes associated with maternal endocrine stress. On the other, their exposure during pregnancy can affect the offspring's metabolic, endocrine and reproductive functions by altering fetoplacental programming. This review highlights the latest development in maternal metabolic and endocrine modulations from exposure to estrogenic mimic chemicals on subcellular and transgenerational changes in placental development and its effects on fetal growth, size, and metabolic & reproductive functions.

## 1 Introduction

Pregnancy hormones such as estrogen, human chorionic gonadotropin (hCG), glucocorticoid, leptin, insulin, prostaglandin, and others are vital to a successful pregnancy. There are several risks associated with pregnancy-related complications, but one among them is the exposure of the mother and fetus to the modulators of pregnancy hormones by endocrine disruptors (1). Endocrine-disrupting chemicals (EDCs) can mimic the body’s hormonal response over life-long exposure. Among these thousand chemicals, endocrine disruptors such as bisphenols (BPs) and phthalates are abundant and present in substances contaminated daily, including food surfaces, cosmetics, and many more. The mechanisms underlying the endocrine stressors during critical development phases on predisposing adult disease risk are evolving (2). Epigenetic modification ensures developmental plasticity that allows adaptive regulation of the developing fetus at the expense of altered placental functions in response to EDC exposure. Normal placentation is the key to a healthy fetus. At the same time, exposure to EDCs can modulate placental functions by altering placental invasion and angiogenesis required for successful placentation (3). Placenta is the active barrier between the mother and fetus and involves waste disposal, gaseous exchange, immunogenic barrier, and hormone production. EDCs can affect placental barrier functions, structure, and stability of gene expression due to maternal exposure during pregnancy that modulate maternal endocrine balance, implantation processes, and fetus organ development (4).

BPA is an estrogenic chemical that disrupts the development programming of reproductive function through changes in gonadal hormones (5). Prenatal phthalate exposure disrupts the hormonal balance in pregnant women and transmits its risks to their offspring (6). In addition, exposure to EDCs predisposes the risk of preterm birth and altered intrauterine growth, gestational length, birth weight, ponderal index, femur length, abdominal circumference, head circumference, and anogenital index in newborns. Scientific investigations have consistently shown the interplay between epigenetics, lifestyle exposure, and the gestational environment. The epigenetic changes due to exposure to EDCs during critical windows of gestation and its effects on gastrulation, fetal organogenesis, and overall growth and development are emerging. The critical window of EDC exposure during pregnancy can disrupt the programming of organ development that could have long-lasting effects. Fetal exposure to these EDCs, such as BPs (bisphenol A, bisphenol S), Phthalates (diethylhexyl phthalate, DEHP and their metabolites) are suspected of altering the programming of normal biological functions, including endocrine imbalance and fertility. This review consolidates pregnancy-linked hormonal balance in response to EDC exposure, the endocrine effects on placentation, epigenetic changes, and maternal endocrine regulation on fetal growth and reproductive development.

## 2 Exposure to endocrine disruptors affects maternal endocrine balance and fetal growth, development, and pregnancy outcomes

Pregnancy is a complex physiological process regulated by various hormones and signalling molecules within the endocrine system. The maternal endocrine system changes during pregnancy to support fetal growth and development that involves changes in several axes, including hypothalamic-pituitary-adrenal (HPA), thyroid, and gonads. The HPA axis is crucial in regulating the maternal endocrine system during pregnancy. The hypothalamus secretes the corticotrophin-releasing hormone, which stimulates the pituitary to release adrenocorticotropic hormone (ACTH). ACTH, in turn, stimulates the adrenal glands to produce cortisol, an essential hormone for fetal development. However, excessive cortisol production can lead to adverse pregnancy outcomes, such as premature birth and low birth weight (7). The thyroid regulates metabolism, growth, and development during pregnancy. The thyroid gland produces two hormones, triiodothyronine (T3) and thyroxine (T4), essential for fetal brain development. The demand for thyroid hormones increases during pregnancy, and the maternal thyroid gland enlarges to meet this demand. In addition, the placenta produces hCG, which stimulates the maternal thyroid gland to produce more thyroid hormones. However, excessive thyroid production can lead to hyperthyroidism, negatively affecting the mother and fetus. Therefore, tight regulation of the thyroid axis is essential in protecting the mother during pregnancy (8).

The reproductive organs undergo significant changes during pregnancy to support fetal growth. The ovaries stop producing eggs, and the placenta takes over the production of estrogen and progesterone, essential for maintaining pregnancy. Placenta does not have all the necessary enzymes to convert cholesterol to estrogen or make progesterone (9). Estrogen and progesterone promote the growth of the uterus and help maintain the pregnancy by relaxing the uterus and preventing contractions. In addition, estrogen promotes the development of the fetal lungs, liver, and immune system. However, excessive estrogen production can lead to adverse outcomes, such as preeclampsia and gestational diabetes. Therefore, tight estrogen regulation is essential for optimum pregnancy outcomes (10).

Placenta acts as an endocrine organ during gestation, secretes several hormones essential for adapting maternal physiology, and transfers nutrients and gases to the growing fetus, thereby supporting fetal development (11). The trophoblast of the developing placenta produces hormones to ensure that the endometrium’s uterus lining can receive them for embryo implantation. As the gestation progress, the placental hormones control maternal physiology to supply adequate oxygen and nutrients to support fetus growth and development. Syncytiotrophoblasts are the cells that produce placental hormones in a tightly regulated manner. Several hormones regulate fetoplacental growth, including glucocorticoid, leptin, gonadotrophin, insulin, insulin growth factor, prostaglandin, and others.

Bisphenols can cross the placenta from maternal blood into the fetal compartment and are detectable in the amniotic fluid and cord blood, indicating exposure to developing fetuses (12). BPA exposure during a critical developmental window can affect trophoblast proliferation, migration, invasion, fusion, apoptosis, and placental morphology. *In-vitro* studies have shown that placental JEG-3 cells treated with low doses of BPA altered steroidogenic genes CYP11A1 and CYP19 (13). In addition, trophoblast cells (BeWo) increased β-hCG secretion and trophoblast cell fusion upon treatment with BPA (14). The exposure to low doses of BPA in pregnant mice from day 1-7 caused degenerative changes in the placenta's trophoblastic giant cells and spongiotrophoblast layers.

Moreover, the placenta's intervillous spaces were narrower in BPA-exposed mice (15). The pregnant CD-1 mice administered with BPA from gD 1 to 11 affected placental angiogenesis and caused necrosis and degeneration of giant cells in early pregnancy (16). In mice, exposure to BPA during preimplantation affects nutrient transport in embryo, preimplantation embryo development, and uterine receptivity (17).

Additionally, the progeny of mothers exposed to BPA was growth-restricted compared to the controls (18). Female rats orally administered with BPA showed reduced lumen diameter, acetylcholine-relaxation, and expressions of endothelial nitric oxide synthase 3 (NOS3), estrogen receptor α (ERα), and peroxisome proliferator-activated receptor ɣ (PPARɣ) in the uterine arteries (19). The low levels of BPA exposure have decreased the trophoblast invasion by altering the balance between the MMPs/TIMP in BeWo cells (20). Similar findings were reported in the *in-vivo* study conducted on pregnant mice exposed to BPA, which exhibited pre-eclampsia-like features like decreased trophoblast cell invasion, increased retention of smooth muscle cells, and a decreased vessel area at the junctional zone of the placenta (21). BPA exposure to the placenta is also reported to alter its metabolic state by altering the levels of GLUT1 expression in the villous explants (22) and placental HTR8/SVneo cells (23).

Increased expression of glucose transporters in the placenta promoted excess glucose transverse to the fetus, induce fetal hyperglycemia, and cause associated developmental defects. Pre- and post-conceptional exposure to bisphenols decreased the area occupied by a spongiotrophoblast relative to trophoblast giant cells within the junctional zone and markedly reduced placental serotonin (5-HT) concentrations. In addition, they lowered the 5-HT GC immunoreactivity (24). BPS-exposed placentas had lower E-cadherin expression, fewer binucleate cells, and higher glial cell missing-1 protein expression. Interestingly, BPA exposed placentas were unaffected, highlighting the intrinsic differences among bisphenol chemicals (25). This evidence suggests that exposure to BPA during pregnancy may adversely affect placental function and fetal development. Thus, due to EDC exposure, fetal development could be impaired due to altered placental response involving its growth, hormonal balance, gene expression, epigenetic modification, and angiogenic and invasive activities.

Placenta is the critical endocrine organ whose formation and development dictate the fate of a successful pregnancy. Placenta plays a vital role during gestation, including implantation of the fetus to the uterine wall, placentation, hormone secretion, uterine artery remodelling, gaseous exchange, transport of nutrients, removal of waste materials, and parturition. Any disruption or imbalances in hormonal production can adversely affect fetoplacental development. Data show that exposure to endocrine-disrupting chemicals like bisphenols A and their substitutions (BPS, BPF, TBBPA, BADGE) affect early development stages. Early developmental involves implantation, trophoblast invasion, and remodelling are modulated by BPA (16, 17, 26–32), BPS (33, 34), BPF (32, 34), and TBBPA (35) exposure. The trophoblast Syncytialization and nutrient exchange across the placenta are affected by BPA (22, 23), and BPS (36) exposure. Feto-placental growth outcomes such as intrauterine and fetal growth, & weight, ponderal index, femur length, abdominal circumference, head circumference, and ponderal index are altered by several EDC exposure, including BPA (23, 24, 37–41), BPS (24, 25, 39, 42), BPF (39), TBBPA (40, 43, 44), and BADGE (45).

### 2.1 Effects of maternal glucocorticoids on the fetoplacental growth and development

Endocrine stress elicits metabolic responses through the centrally coordinating neuroendocrine by activating the HPA-axis through hormonal signalling, leading to the systemic release of glucocorticoid (GC) from the adrenal (kidney) in humans (46). GCs act as maturation signal to sense EDC exposure and regulates intrauterine programming of adult physiological phenotype. Excess exposure to endogenous GCs activates maternal HPA by augmenting nutritional and stress response during pregnancy and increases fetal GC concentration. Fetal GC can be elevated independently of the mother to compensate reduced supply of nutrients and oxygen or by the changes in the placental activity of the 11HSD enzymes. The placenta produces an enzyme called 11-beta hydroxysteroid dehydrogenase type 2 (11β-HSD2), which inactivates cortisol in the placenta and prevents it from reaching the fetus.

Maternal GC affects the growth and development of the placenta by modulating surface areas for nutrient exchange, formation of fetal blood vessels, and expression of nutrient transporters across the placenta. Glucocorticoid receptor (GR) is ubiquitous in the cellular system and highly expressed in the placenta. Maternal GC has multiple ways of controlling fetoplacental growth, including its effects on the placental vascularization (47), expression of nutrient transporters in the placenta (48), cardio-metabolic maturation of the newborn heart (49), birth weight of the offspring (50), change in body fat deposition and body composition of the primate offspring (51), and others. Excess GC exposure during early fetal life in humans results in glucose intolerance later in life due to the reduced ability to secrete insulin at adult age (52). Placental 11β-HSD2 is a key GC metabolizing enzyme that controls fetal GC levels and mineralocorticoid receptors specificity in the kidney. In the kidney, the enzyme acts as a receptor-modulator of metabolically active GCs, such as cortisol (human) and cortisone (animal), to convert to their derivatives. In the physiological state, maternal GC levels are relatively higher than fetus but protected from excess GC exposure by the placental 11β-HSD2 enzymes. Inhibition or mutation of 11β-HSD2 is reported to lead to low birth weight (LBW) (53, 54). During human pregnancy, cortisol levels are elevated in the maternal circulation (55). However, the placental HSD11β protects the fetus from exposure to high levels of maternal GCs (56). The reduced placental activity and expression of HSD11β_2_ correlates with lower birth weight (57, 58), and dysregulated placental HSD11β_2_ expression is reported in intrauterine growth restriction (57, 59). Therefore, optimal placental HSD11β balance is critical for the organ growth of the fetus and its maturation.

### 2.2 Placental leptin and its roles in fetal growth

Leptin is a fat cell-produced hormone that regulates satiety, signalling, and energy balance (60). Leptin (16KDa) is the novel placenta-derived hormone in humans (61); it plays an endocrine role in controlling maternal satiety, energy metabolism, and fat metabolism and supports trophoblast proliferation and survival (62). Leptin produced by the placenta and adipose tissue has a similar size, charge, and reactivity, but their upstream regulations differ. The human placenta expressed a comparatively higher leptin mRNA and protein than rodents (63). Both placental and maternal circulatory leptin increases with gestation, indicating leptin’s role in maintaining pregnancy.

Expression of the leptin gene in placental vascular endothelial cells in contact with fetal blood first highlighted its potential role in placental angiogenesis. Leptin is co-localized with syncytiotrophoblasts on the maternal side in humans. In contrast, it is co-localized at both the maternal side and labyrinth of the cytotrophoblast of the developing fetus in rodents. The leptin receptor gene (OB-R) in mouse placenta has different variants, including signalling receptor (OB-Rb), transporter receptor (OB-Ra), and soluble receptor (OB-Re). The placental leptin content is regulated by changes in maternal hormones like progesterone and cortisol, which can affect leptin production in maternal adipose tissue in humans (64). A positive correlation exists among circulatory leptin, hCG, and estradiol concentration in pregnant women (65). Leptin and hCG act as a potential regulators of angiogenesis by controlling VEGF secretion at the maternal-fetal interface (66). The leptin receptor was upregulated by hypoxia in placental cells (67). Placental leptin is a biomarker of placental hypoxia in severe preeclampsia (68). Maternal fat deposition increases during pregnancy, but the mother’s BMI does not correlate with the gestational increase in circulatory leptin, indicating the contribution of leptin from the placenta, not from maternal adipose tissue (69). The leptin binding to soluble leptin receptors secreted by the placenta increased circulatory leptin and prevented leptin from binding to signalling receptors, leading to leptin resistance (70). The soluble receptor of leptin concentration was decreased rapidly in the later gestation, around 20-30 weeks (71), suggesting leptin’s role in the catabolic phase of pregnancy in regulating maternal appetite and fat metabolism. It was proposed that leptin could promote placental development by stimulating angiogenesis (72). Although leptin’s role in placental angiogenesis is unclear, *in vitro*, data showed that leptin stimulated tube formation independent of VEGF and upregulated expression of genes associated with angiogenesis in the placental cells (73). In addition to its angiogenic effects, placental leptin showed anti-inflammatory roles by producing excess in GDM and PE to counter the effects of proinflammatory cytokines (74). Leptin is essential in fetoplacental growth and development as cord blood leptin positively correlates with birth weight (75). Maternal hyperglycemia in insulin-dependent diabetes during pregnancy enforces the fetal pancreas to release insulin that increases fetal and placental leptin concentration in paracrine and induces fetal overgrowth and macrosomia (76). Maternal hyperglycaemia was associated with DNA methylation of the leptin gene, which could risk childhood obesity (77). Placental leptin gene expression was reduced due to BPA exposure (38). Leptin level was increased in BPA-exposed overweight mice in the non-fasting state, indicating the involvement of leptin resistance in these animals (78). BPS exposure potentiated the obesity induced by a high-fat diet and was linked with a higher fat mass, food intake, and hyperleptinemia (79).

Overall, the placenta regulates its growth by its leptin in an autocrine manner since cord blood leptin concentration correlates with placental size. Again, placental leptin executes paracrine regulation of maternal energy balance by increasing its concentration later in the pregnancy, thus sustaining fetal growth and development.

### 2.3 Maternal chorionic gonadotrophin and placental trophoblast functions

Chorionic gonadotrophin (hCG) is produced by the placenta. During early pregnancy, trophoblasts secrete hCG, as the first placental hormone (80). hCG promotes the production of ovarian progesterone and estrogen from the corpus luteum. Later, the placenta takes over the progesterone production as steroid biosynthesis occurs throughout the gestation (81). hCG is a glycoprotein with α and β subunits linked by a non-covalent bond. The α-subunit is encoded in chromosome 6 and is similar to other glycoproteins such as follicle-stimulating hormone (FSH), luteinizing hormone (LH), and thyroid-stimulating hormone (TSH). The β-subunit is encoded by chromosome 19 and is different for each hormone. hCG exists in different isoforms, such as classical hCG, hyperglycosylated hCG, and the free β unit of the hyperglycosylated hCG (82). The hCG levels peak during the first trimester, later decrease, and remain at basal levels throughout pregnancy (83). The biological functions of hCG include angiogenesis, vasculogenesis (84), and differentiation of cytotrophoblasts into syncytiotrophoblasts (85, 86). A retrospective cohort found that in the first trimester, low free β-hCG levels significantly increased the risk for preterm birth, LBW, intrauterine growth restriction (IUGR), and low APGAR score (87). Immunostaining of placental tissues reveals α-hCG staining rates decreased significantly in IUGR cases over the control (88).

In contrast, the high free β-hCG group had a significantly decreased risk of GDM and preterm birth. In the second trimester, low and high β-hCG had substantially higher risks of the most common adverse outcomes, i.e., spontaneous abortion, IUGR, and preterm birth (87). Moreover, free β-hCG was associated with an increased risk of spontaneous fetal loss (89). Serum β-hCG levels of the missed abortion were lower than those of the control group. Decreased hCG production in early pregnancy down-regulates the expression of the VEGF-MEK/ERK signal pathway, ultimately reducing the angiogenesis (90).

Moreover, studies also show a relationship between gestational trophoblastic disease and hCG. Thus, maternal hCGs involved in several gestational events during pregnancy while their abnormal levels are associated with adverse pregnancy outcomes. Higher exposure to BPA and phthalates was associated with a lower hCG in pregnancy, suggesting altered production or secretion of hCG by the placenta (91).

### 2.4 Insulin, placentation, and nutrient transport

The peptide hormones such as insulin, IGF1, and IGF2 perform mitogenic and metabolic effects by specifically binding to their surface receptors in target tissues. In normal physiology, insulin and IGFs bind to their receptor, such as IR or IGF1R, in a rapidly growing embryonic tissue (92), thereby regulating placental growth and transport, trophoblast invasion, and placental angiogenesis. Insulin (1ng/ml) stimulated angiogenesis by increased KDR expression in the extravillous HTR8/SVneo cells (93), which is required for vessel formation to promote angiogenesis in the placenta. Maternal diabetes could result in hypervascularization and excess angiogenesis in the placenta due to dysregulated insulin, growth factors, and excess pro-angiogenic effects of insulin in the endothelial cells (94). Placental IGF1 and IGF2 expression are regulated by compartment and gestational stages. For example, IGF2 was not detected at term but was highly expressed in the first trimester villous and extravillous cytotrophoblasts, indicating its significant roles in the early embryonic development involving the placentation (95–97). During the early trimester, these IGFs regulate the uterine-trophoblast invasion (98), migration (99), and placental LPL activity (100). Moreover, IGFs regulates FABP4 expression (101), transplacental glucose and amino acid transporters (102) via activation of mammalian target of rapamycin (mTOR) signalling (103), and increased basal membrane GLUT1 content and trans epithelial glucose transport (104). Several factors, including mTOR signalling, regulate the nutrient transport mechanism. In trophoblast cells, mTOR senses the nutrient signal for maternal supply and fetal demand. mTOR senses various maternal endocrine signals (such as insulin, leptin, and others) to regulate fetal growth by activating gene transcription and protein expression, resulting in modulated expression of nutrient transporters. The maternal hormones such as insulin, leptin, and IGF1 stimulate, while hypoxia and adiponectin downregulate the mTOR signalling (105). Protein restriction during pregnancy affects insulin signalling and glucose metabolism in the muscle tissue of the offspring by involving mTOR (106). Pregnancy complications associated with obesity and endocrine stress due to EDC exposure can affect the mTOR pathway, leading to altered placental nutrient transporter efficiencies and influencing the fetus and development.

### 2.5 Prostaglandin and placental trophoblast development

Prostaglandins (PGs) are lipid-derived hormone-like molecules that modulate several fetoplacental growth and development functions. Unlike hormones produced by endocrine glands released into the bloodstream, prostaglandins are produced by specific tissues at the site of action. Prostaglandins are produced from arachidonic acid, 20:4n-6 (ARA), by phospholipase A2, followed by cyclooxygenase (COX). A higher ARA level in the early placenta supports prostaglandin’s roles in early development, i.e., decidualization, vascularization, angiogenesis, organogenesis, and others (107–110). Moreover, ARA does not accumulate in the placenta near term since its derivative can trigger parturition. In contrast, the precursor of the vasorelaxant and anticoagulant PGE1 levels are increased at period, facilitating blood flow to support fetal growth spurt. Prostaglandin E2 (PGE2) is abundantly produced by the decidua that helps the migration of first-trimester extravillous trophoblast by increasing the intracellular calcium concentration (111). Upregulating PGE2 production and PGE2 receptor expression associated with increased levels of leukemia inhibitory factor favors EVT invasion during the placentation (112). Early stages of spiral artery remodeling mimic angiogenesis, are facilitated by several cytokine-producing cell types and therefore remain critical for early placentation that involves EVT (113, 114). PGE2 is involved in angiogenesis (115) by acting on various cell types that produce proangiogenic factors, such as VEGF, CXCL1, and bFGF, which act on target endothelial cells to promote the angiogenesis (116). Multiple signaling mediators by the various PGE2 receptors facilitate the PGE2-induced angiogenic response. PGE2 stimulated *in vitro* angiogenesis comparable with VEGF in trophoblast cells. However, PGE2-induced angiogenesis was inhibited by COX2 inhibitors. In contrast, VEGF-induced tube formation was the least affected (110), indicating that PGE2 may have an independent effect on angiogenesis in the placenta. Another PG, like PGF2α levels, increases during the window of implantation in the uterine lumen of humans and other mammals, suggesting its role in embryo implantation. PGF2α reported stimulating invasion and migration of the human trophoblast HTR8/SVneo cells (117). PGF2α increased decidual gelatinolytic activity in fetal membranes since its inhibition helps arrest the labor (118). Prostaglandins and their derivatives perform multiple roles associated with fetoplacental growth and development, including embryonic implantation, vascularization, angiogenesis, trophoblast invasion, migration, and labor.

## 3 Roles of placental steroid hormones in pregnancy

Placenta acts as the primary organ for steroid hormone synthesis during gestation. Placenta contains all the necessary enzymes for steroid biosynthesis. The cytochromes P450s (CYPs) and the hydroxysteroid dehydrogenases (HSD) are the enzymes in the placenta where hormonal biosynthesis takes place from the maternal cholesterol (119). The placenta converts cholesterol into pregnanolone (PREG) and finally to progesterone by a series of enzymatic reactions. In addition to the placenta, fetal tissues can convert PREG to dehydroepiandrosterone sulfate (DHEAS) and finally to 16α-OH-DHEAS. Hydroxylated DHEAS and DHEAS again enter the placenta and eventually transform into androstenedione and testosterone. In the placenta, the aromatase enzyme (CYP19A1) converts androstenedione and testosterone into estrogen [estrone (E1), estradiol (E2), or estriol (E3) (119). Progesterone predominantly mediates its actions by binding to the nuclear receptor (PR-A and PR-B) and membrane-bound receptor causing non-genomic actions.

High progesterone levels are correlated with the development of glucose abnormalities in pregnancy. Recent studies have shown that progesterone receptors knocked out in female mice (PR-/-) but not male had lower fasting glycemia than PR+/+ mice and showed higher insulin levels on glucose injection. Moreover, pancreatic islet cells were larger and secreted more insulin due to increased β-cell proliferation (120). Placental estriol, estradiol, and progesterone levels were lower in preeclampsia's placental tissues than in healthy pregnant women (121). In preeclampsia, a severe hypertensive disorder of pregnancy, the sirtuin1 expression was lower in serum and placenta. Progesterone alleviates preeclampsia-like symptoms mediated by SIRT1 deficiency (122). Progesterone prepares the uterus for pregnancy and prevents preterm births by inhibiting the contraction of uterine muscles. Vaginal progesterone was associated with a significant reduction in the risk of preterm birth (<33 weeks of gestation), spontaneous preterm birth, and admission to the neonatal intensive care unit in pregnancies with short cervix (123). Vaginal progesterone administration significantly decreased the risk of preterm birth, neonatal death, composite neonatal morbidity, and mortality in women with twin gestation and short cervix (124).

As mentioned, the enzyme aromatase converts androgen precursors from fetal and maternal adrenal glands into estrogen in the placenta. The classical estrogen receptors (ER-α & β) and the plasma-bound G protein-coupled ER (GPR30/GPER) are expressed in the uterine artery endothelial cells (UAECs) and smooth muscle of the uterus, mediating their actions through genomic or non-genomic pathways. Activation of ERs by estrogen enhances UAEC's nitric oxide production, thereby causing uterine vasodilation during pregnancy (125). Knockout of ER-α in the female mice decreased litter size and maternal nurturing behavior (126). Estrogen receptor-β is involved in implantation and parturition in the mature uterus (127). Insulin resistance associated with hyperestrogenemia occurs in GDM and PCOS. Estradiol can bind to insulin and may interfere with binding to its receptor, causing insulin resistance (128). Recent studies have shown a possible link between estradiol and glucose homeostasis. Cord blood estradiol in the GDM was significantly lower than in the control group.

Moreover, the cord blood estradiol concentrations were negatively correlated with birth weight (129). Estrogens are well known to modulate angiogenesis in both physiological and pathological conditions. Exogenous estrogen stimulates cell proliferation and angiogenesis in the pregnant ewe UAECs (130). Female ovariectomized ER knockout mice showed impaired angiogenesis, suggesting the role of ER in the angiogenesis (131). As estrogens promote angiogenesis and vasodilation, preeclampsia may be associated. Plasma 17β-estradiol levels were significantly reduced in severe PE subjects (132). Similar outcomes were found in patients with mild to severe PE (133). Placental tissue levels of estriol and estradiol were significantly lower in PE compared to healthy pregnant women (121). Overall data indicate the role of estrogen in the pathophysiology of preeclampsia, which could be caused due to aromatase deficiency in the placenta.

## 4 Endocrine modulating factors during pregnancy and gestational disease risks

Several studies mentioned in this section have reported a relationship between placental hormones and the development of IUGR. Low hCG concentrations during the late first trimester were associated with decreased fetal growth, birth weight, and small for gestational age (SGA) (134). A similar study was conducted on 9450 singleton pregnant women. SGA was associated with low maternal serum levels of pregnancy-associated plasma protein-A (PAPP-A), free β-hCG, and slow early fetal growth (135). β-hCG levels were significantly decreased in SGA cases compared to the control (136). Sex hormones also regulate IUGR. 17-β Estradiol (E2) is critical to a physiological pregnancy and synthesized by the conversion of androgens by the CYP19A1 gene. Maternal E2 level in plasma was significantly decreased in the IUGR. Although the placental expression of CYP19A1 was considerably higher than in control (137), serum levels of estriol (E3) were significantly lower in the IUGR (138). Moreover, the estrogen-related receptor gamma expression was reduced in the FGR placenta compared to the control (139).

Gestational diabetes mellitus is characterized by spontaneous hyperglycemia during pregnancy. The Hyperglycaemia and Adverse Pregnancy Outcome (HAPO) study found that maternal hyperglycemia independently increased the risk of preterm delivery, cesarean delivery, infants born large for gestational age, and neonatal hypoglycemia (140). Although GDM is a pregnancy disorder, it resolves following delivery but can have long-term health consequences on maternal and fetal life. Risk factors include obesity, diabetes, a sedentary lifestyle, genetics, and advanced maternal age. The primary pathophysiological condition in GDM is β-cell dysfunction. It is caused by an inability of β-cells to sense blood glucose concentration and release insulin adequately. Due to this condition, high blood glucose prevails, leading to hyperglycemia. Glucose is the primary energy source for both placenta and fetus development. As glucose is always required, the placenta expresses insulin-independent glucose transporter GLUT1. GLUT1 plays a role in implantation and angiogenesis. The expression of GLUT1 on the basal plasma membrane of the placenta is positively correlated with birth weight (141). However, the expression and function of GLUT1 are down-regulated in pre-eclampsia and IUGR (142).

Moreover, insulin-stimulated glucose uptake of the first-trimester trophoblast cells, HTR8/SVneo, is partially mediated via GLUT1 (93). Trophoblasts and endothelial cells of the placenta also express insulin receptors, which can be activated by insulin and alter the placental metabolism of nutrients (143). Higher hCG levels in early pregnancy are associated with a lower risk of GDM (144). In contrast, elevated hCG levels were especially predominant among women who developed GDM (145). The relationship between hCG and GDM was inconsistent and varied with the duration of pregnancy. Estradiol is an essential mediator of glucose homeostasis. *In-vivo* studies have shown that aromatase knockout mice (ArKO-/-) develop glucose intolerance and insulin resistance. Treatment of ArKO males with 17β-estradiol improved the glucose response (146). Cord blood estradiol was significantly lower in GDM than in control. Moreover, cord blood estradiol was negatively correlated with birth weight. Estrogen protects pancreatic β-cells from apoptosis and prevents insulin deficiency (147). A recent study has also shown a relationship between progesterone and GDM. Compared to controls, GDMs have significantly lower progesterone at weeks 10-14 (148).

Hyperglycemia in mothers can lead to macrosomia due to the excess glucose available to the fetus via the placental glucose transporter GLUT1. In addition to β-cell dysfunction, insulin resistance and several maternal hormones produced by adipose, liver, and placenta can develop GDM. Gestational hCG levels are associated with predictive risk with the development of GDM but inconsistently.

## 5 Endocrine-disrupting chemicals induce endocrine dysfunction and fetal programming of development and reproductive disease

Endocrine-disrupting chemicals interfere with the typical endocrine system and produce adverse effects. EDC was thought to exert its action primarily through nuclear hormone receptors such as ER, androgen receptors (AR), thyroid receptors (TR), and retinoid receptors. However, recent data suggest that EDC can also act on non-nuclear and orphan receptors. EDC can be synthetic or natural chemical compounds. Synthetic chemicals are used as plasticizers [bisphenols, phthalates], pharmaceutical agents [diethylstilbestrol (DES)], industrial solvents [polychlorinated biphenyls (PCBs), polybrominated biphenyls (PBBs), dioxins], agrochemicals [methoxychlor, chlorpyrifos, dichlorodiphenyltrichloroethane (DDT)], fungicides (vinclozolin)]. Natural chemicals include phytoestrogens such as genistein and coumesterol.

**Figure 1:**
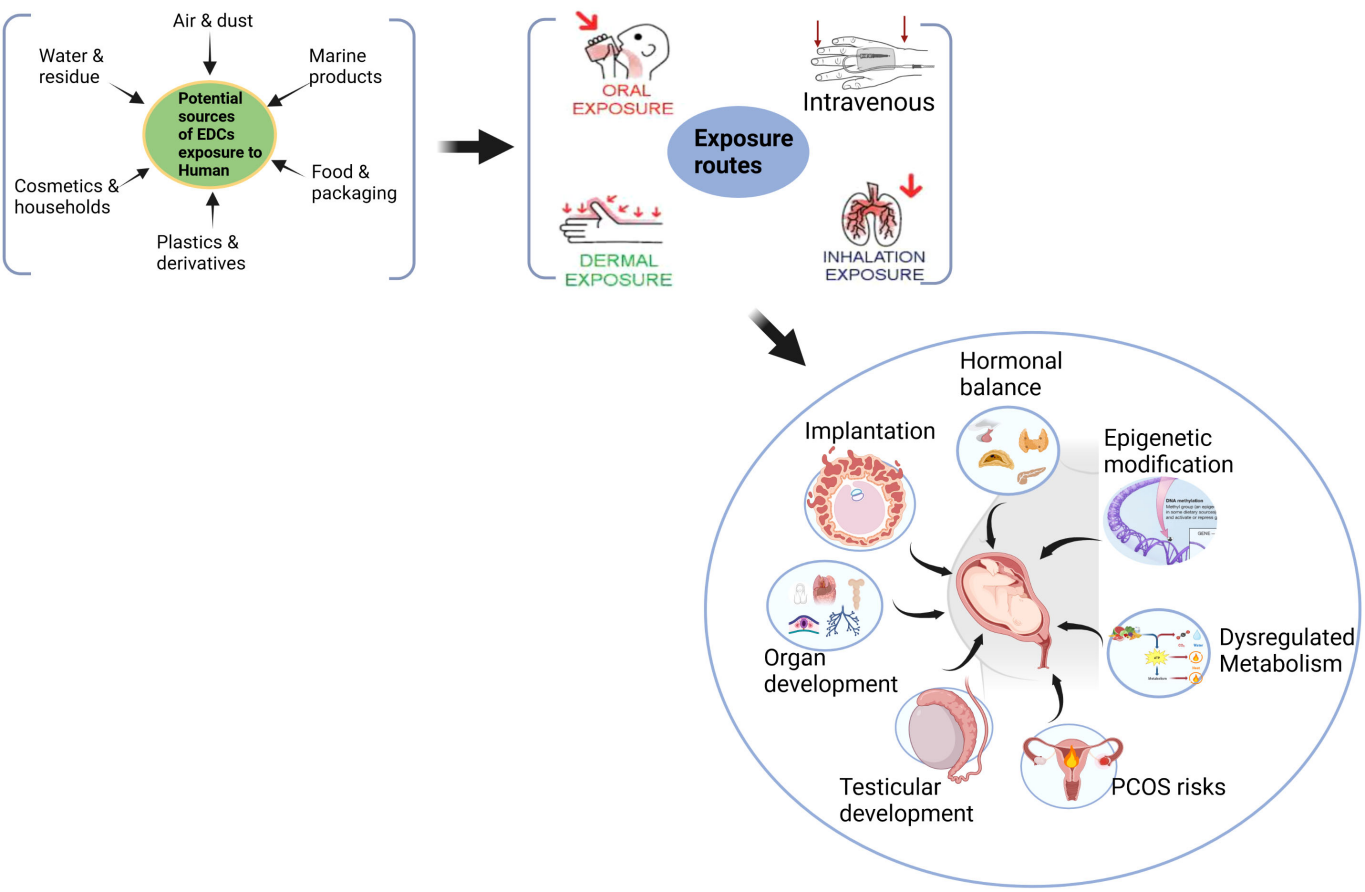
Maternal exposure risk of EDCs in modulating fetoplacental development that they might risk programming adult metabolic and reproductive diseases.

Humans are exposed to EDC by various routes such as ingestion, inhalation, and transdermal. EDCs have been detected in maternal blood, amniotic fluid, and cord blood (149). The EDC’s influence on maternal response may program several adult diseases, particularly during gestation. **Figure 1** describes the life course exposure risk of EDCs on the diverse functional effects in modulating fetoplacental development that are mediated by maternal effects spanning from hormonal imbalance, epigenetic modification, dysregulated metabolic responses and risks of gonadal and reproductive functional deformities in the offspring.

**Figure 2:**
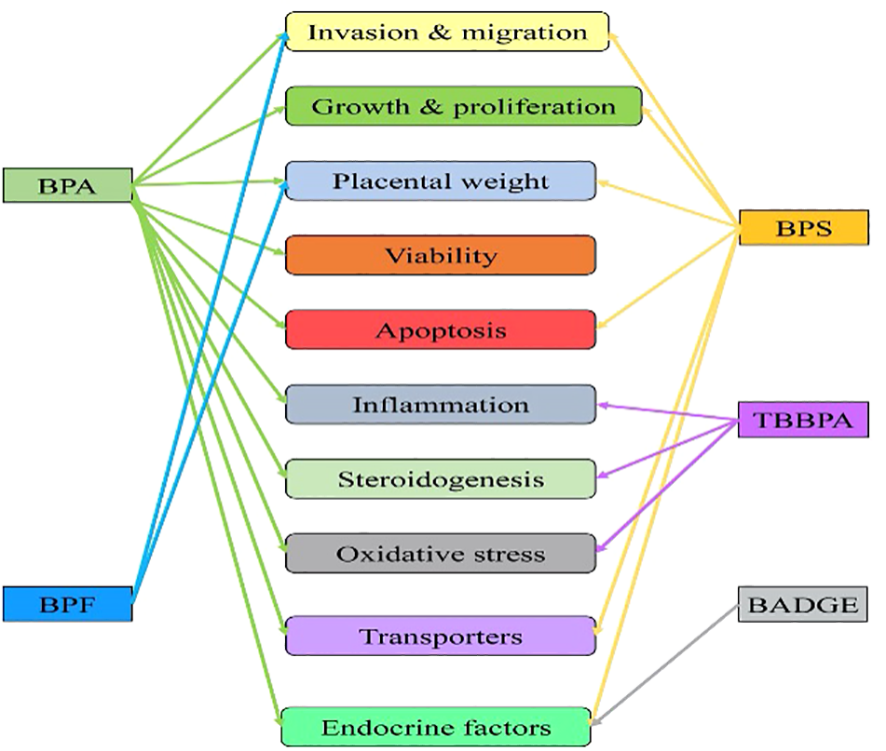
Influence of endocrine-disrupting chemicals (bisphenols) exposure on trophoblast invasion, development, and placental functions. Single-headed arrow indicates an independent study reported so far available from the PubMed database. BPA, Bisphenol A (23, 24, 26–28, 38); BPS, Bisphenol S (25, 33, 36, 42, 150, 151); BPF, Bisphenol F (34, 39); TBBPA, Tetrabromo Bisphenol A (35, 40, 43, 152, (44); BADGE, Bisphenol A diglycidyl ether (45).

Adults can perform rapid first-pass metabolism to convert these into biologically inactive metabolites and rapidly clear them in the urine. Still, the unborn developing fetus and placenta do not have the enzymatic machinery to catabolize these compounds. As these chemicals cross the placenta, EDC threatens the unborn fetus. Placental trophoblast invasion, proliferation, and its several functions are affected due to exposure to bisphenol A (BPA) and their substitution chemicals (**Figure 2**). Research has shown that EDC exposure during the critical developmental window can have long-term health impacts on the fetus and its later adult life (2). Moreover, successful pregnancy depends on the physiological secretion of several hormones. Any changes in the hormonal milieu during development can cause pregnancy-related complications and diseases.

Humans are constantly exposed to synthetic EDCs such as bisphenols and phthalates. The plastic-derived chemicals such as BPA and its substitute bisphenol S (BPS) are the major endocrine disruptors exposed ubiquitously since it mimics hormones and modulates the endocrine system, affecting the metabolic outcome. The food contaminated with packaging and chemical agents mixed with bisphenols is continuously exposed in micron levels through leaching, especially plastic-derived residual contaminants (153). Semisynthetic and plasticizer-laden food surfaces (154) are exposed to heat and change in pH, resulting in chemical hydrolysis of polyester bonds, releasing chemicals directly exposed to living systems (155). BPs are absorbed through the skin, and first-pass metabolized to their conjugates, resulting in a greater endocrine risk to human disease burden (156).

BPA is the most abundant in consumer products, including food and beverage containers, medical devices, toys, and thermal receipts. BPs are detected in human blood irrespective of gender. However, the findings on gender-specific exposure to BPA are inconsistent, as studies have shown that BPA concentrations were higher in men than women (157, 158). At the same time, others found equal concentrations of BPA in both males and females. The discrepancies in the data could be multifactorial, including but not limited to age, sample size, geographical location, and technique. The concentrations of BPA detected in the cord blood are closely related to levels in maternal blood, indicating that BPA readily crosses the placenta. Epidemiology and clinical evidence of EDCs exposure suggest a profound association between the exposure of BPA and its analogs during pregnancy and altered fetal and neonatal growth outcomes, including intrauterine growth, gestational length, birth weight, ponderal index, femur length, abdominal circumference, head circumference and others (**Table 1**).

**Table 1:** Epidemiology and clinical evidence of bisphenol exposure on fetal and neonatal growth outcome.

EDCs	Subjects	Sample types	Assays	Key findings	Ref.
BPA and BPA-glucuronide	Mother-infant pairs (n=60)	Maternal serum and cord blood	Bisphenol and steroid hormone	-Positive relationship between cord blood estradiol, BPA and maternal BPA levels. -Cord blood testosterone from male infants showed a negative correlation with maternal BPA	(159)
BPA	Overweight (n=26) and age-match normal weight pregnant women (n=32)	Placenta	GLUT1 and GLUT4 in placental explants exposed to BPA (1 nM and 1 μM)	-↑ GLUT1 expression in normal weight -↓ GLUT1 expression in overweight -GLUT4 expression lower in the overweight explants	(22)
BPA	Mother- child pairs in third trimester (n=788) and neonate (n=366)	Urine	BPA exposure & fetal and neonatal growth outcome	-Negative correlation between BPA exposure and intrauterine linear growth -Positive association with volume growth during childhood	(160)
BPA and phthalates	Mother-child pairs (n=488)	Urine	Prenatal BPA and phthalate exposure, fetal growth, and birth outcomes at 12, 20, and 34 weeks of pregnancy	-Prenatal phthalate metabolite (MBzP) exposure positively associated with femur length at 20-34 weeks and birth weight among boys. -Mono-n-butyl phthalate (MnBP) negatively associated with head circumference at 12-20 weeks.	(161)
BPA	Pregnant women (n=219)	Urine	BPA exposure, fetal growth, and birth outcomes	-Women with creatinine-normalized BPA>4.22 μg/g had lower growth rates for fetal weight and head circumference than women with lower creatinine-normalized BPA	(162)
BPA	Mother-new born pairs (n=97)	Maternal and umbilical cord blood	BPA exposure and birth outcomes	-Elevated risks of low birth weight, short gestation, and altered secretion of leptin and adiponectin observed in male newborns	(163)
BPA, BPS, and BPF	Pregnant woman before delivery (n=1197)	Urine	Urine bisphenol and fetal growth parameters	-Maternal urinary BPA and BPF negatively related to birth length and positively associated with ponderal index. -BPS exposure associated with short gestational age only in girls.	(164)
BPA, BPS, and BPF	Pregnant women third trimester (n=322)	Urine	Urine bisphenol and fetal growth parameters	-Gender difference in the association of maternal urinary BPA concentrations and fetal head circumference. -Maternal urinary BPF showed an inverse and positive associations with abdominal circumference and femur length respectively	(165)
BPB, BPF, BPS, TBBPA (tetrabromobisphenol A)	Mother-infant pairs (n=2023)	Serum	Serum bisphenol and birth size	-Serum BPA and TBBPA negatively correlated with birth weight -Higher BPF was associated with decreasing birth weight and ponderal index.	(166)
BPA, BPS and BPF	Pregnant women (n=845)	Urine	Urine bisphenol and size at birth	-Urinary BPF and BPS positively associated with lower birth weight, birth length, or ponderal index	(167)
BPA, BPS and BPF	Pregnant women (n=1379)	Urine	Urine bisphenol and birth outcomes	-BPS exposure associated with larger fetal head circumference, higher weight and lower risk for small size for gestation age	(168)
BPA, BPS and BPF	Twin-pregnant women (n=289)	Urine	Urine bisphenol and birth outcomes differences in twins	-Urinary BPA positively associated with the within-pair birth weight and birth length differences across pregnancy trimesters	(169)
BPA	Pregnant women (n=120)	Urine and serum	Urinary bisphenol, serum β-hCG and anthropometry	-No significant association existed between BPA and β-hCG with birth outcomes	(170)
BPS	Pregnant women (n=985)	Urine	Urinary BPS and birth outcomes	-Higher maternal urinary BPS associated with increased gestational age	(171)

“↑” and “↓” indicate up and down-regulated respectively.

Trans maternal exposure to BPA in non-obese diabetic mice increased the severity of insulitis and the incidence of diabetes in female offspring (172). Perinatal exposure to BPA alters the expression of genes involved in β-cell growth regulation, incrementing β-cell mass/area and β-cell proliferation during early life. Excess insulin production in early life may impair glucose metabolism in later life (173). Additionally, the metabolic changes caused by *in-utero* BPA exposure were found to be inheritable. i.e., maternal exposure to a low dose of BPA impaired insulin secretion induced islet inflammation and increased β-cell death in the offspring. Surprisingly, subsequent generations (174) and third-generation (175) inherited these changes, indicating that BPA-induced epigenetic modifications were inherited.

Although excess GC exposure can modulate fetus growth and development, little is known about how the GC system is vulnerable to BPs exposure, particularly during pregnancy. Data suggest that BPs significantly inhibited HSD11β2 activity in humans and rats, possibly by steroid binding site, as evidenced by docking analysis (176). It is well established that BPA exposure modulates cellular function by their estrogen agonist or androgen antagonist function. Still, their preference for binding GR or ER in the placenta is unknown. BPA may mediate its effects via a non-classical ER pathway without GC, where ER antagonists inhibit BPA-induced adipogenesis but not GR antagonists (177). BPA at lower concentrations increased the expression of both protein and mRNA of HSD11β_2_ in placental trophoblast cells (38). Our recent data showed that prenatal BPs exposure lowered HSD11β2 expression in the rat testis (178), indicating reduced protection against cortisol-mediated stress induced by bisphenols in these offspring.

BPA possesses pseudo-estrogenic and anti-androgenic effects that might interfere with the developmental programming of the male reproductive system in the offspring. Prenatal exposure to BPA and BPS at much lower concentrations than TDI, during gestational days 4 to 21, alters testis weights, histology, increased testicular inflammation, and oxidative stress in the adult offspring. Moreover, sperm DNA damage and DNA methylation were also increased in both BPA and BPS-exposed adult offspring (178). Exposure to BPA and its analogs bisphenol B (BPB), bisphenol F (BPF), and bisphenol S (BPS) during gestation resulted in decreased testosterone secretion, sperm production, and histological changes in the reproductive tissues of adult SD male rats (179). BPA exposure during pregnancy and lactation can also affect epididymal sperm count and sperm motility (180). CD-1 mice exposed to bisphenols, dysregulated serum estradiol-17β, testosterone, expression of steroidogenic enzymes, increased mRNA levels of DNA methyltransferases, and histone methyltransferases in F3 adult testis (181). Similar results were found with BPA and its analogs on female reproductive functions in mice. Early life exposure in mammals affects early oogenesis and follicle formation in the fetal ovary (182, 183). Bisphenol exposure in the F0 generation accelerated the onset of puberty and exhibited abnormal estrous cyclicity in F3 females (184). Gestation and lactation-specific BPA exposure decreased sex hormones and reduced the number of tertiary ovarian follicles in the female offspring (185). BPA exposure affected placentation and exhibited preeclampsia-like phenotype in pregnant mice (21). Exposure to BPA was also associated with a risk of preterm birth (186).

Growing evidence suggests that BPA and its substitute BPS have distinct action modes in different tissues. e.g., *in-utero* exposure to BPA altered endogenous long-chain fatty acid metabolism in the testis of adult offspring, thereby affecting sperm maturation and quality. However, no changes were observed in the testis of BPS-exposed offspring (187). The bisphenols interfere developmental programming of the reproductive system in the offspring (178) since fetuses do not equip with enzymatic machinery to catabolize these compounds (2). Thus, *in-utero* exposure to BPA during development can program the fetus for endocrine-related diseases in later life.

## 6 Endocrine-disrupting chemicals and endocrine risk of pre-pregnancy

Female infertility is a rapidly evolving endocrine disorder in pre-menopausal women, with an estimated prevalence of 5%-18% globally (188). Often it remained underdiagnosed and became one of the leading causes of female infertility. The risk of failed pregnancy can be associated with metabolic, reproductive, and endocrine features such as anovulation, hyperandrogenism, arrested folliculogenesis, infertility, polycystic ovaries, type 2 diabetes, and obesity (189). Dysregulated hypothalamic‐pituitary‐ovary (HPO) axis, followed by hyperandrogenism, increased serum luteinizing hormone, and metabolic disturbance, contribute to the complex etiology of PCOS (190). Despite studies, the pathophysiological cycle of hyperandrogenism, hyperinsulinemia, and anovulation in PCOS remains unclear. Studies pointed out that exposure to common household plastics containing chemicals could associate with risks of PCOs in humans.

BPA levels were significantly elevated in women with PCOs (191). Similarly, adolescent girls with PCOS had markedly increased serum BPA than controls, and BPA was correlated considerably with androgen (192). Thus, BPA may play a potential role in the etiology of PCOS, specifically a significant contributor to ovarian hyperandrogenism observed in women with PCOS. In addition to hormonal changes, BPA can exert its effects through epigenetic modifications. BPA can change DNA methylation or alter histone modifications without altering the nucleotide sequence of the genes. BPA altered stress-related promoter DNA methylation in placental cells (27). *In-utero* BPA exposure decreases the CpG islands in the promoter and intronic region of the HOXA10 gene, which is involved in uterine organogenesis (193). Prenatal or neonatal exposure to BPA causes an increase in levels of androgen-hyperandrogenism, one of the hallmarks of PCOS. Hyperandrogenism promotes epigenetic modifications of histone deacetylase 3 (HDAC3), peroxisome proliferator-activated receptor gamma 1 (PPARG1), and nuclear corepressor 1 (NCOR1) genes in granulosa cells of PCOS women (194). BPA exposure could dysregulate endocrine balance by changing the expression of several genes, such as CYP11A1, GnRH, AdipoQ ESR1, and StAR, whose disruption is commonly observed in the pathogenesis of PCOS (195).

The endocrine disruptor BPA can mimic estrogen to interact with its receptor. BPA exposure modulated steroidogenic enzymes' expression in the ovary, lowering aromatase expression and estrogen production in granulosa cells. BPA is reported to stimulate androgen synthesis in ovarian theca-interstitial cells. Moreover, BPA concentration was significantly higher in PCOS patients' follicular fluids. BPS is structurally similar to BPA, where sulphonyl groups substitute phenolic groups. *In utero*, BPS exposure also disrupted female reproductive development by changing the estrogen-responsive gene expression in the uterus and ovary (196). Whether exposure to bisphenols is causally related to developing PCOs risk is not established yet. However, BPA could be a causal factor for PCOS because it mimics the hormone estrogen's function (197). BPA (500 μg/kg/day) exposure causes irreversible alteration in the hypothalamic-pituitary-gonadal axis in rats, leading to anovulation and infertility (198).

Subcutaneously injected with BPA from postnatal days 1 to 10 resulted in the development of the PCOS morphology after 4 to 5 months. Serum testosterone and estradiol were elevated, with an irregular GnRH pulsatility (199). In a similar study, SD female offspring perinatally exposed to 1.2 mg/kg bw/day BPA from gD 6 till lactation exhibited increased body weight, altered patterns of estrous cyclicity, and decreased plasma LH levels (200). Prenatal exposure to BPA in CF-1 mice significantly reduced the days between the vaginal opening and the first oestrus (201). Similarly, ICR mice prenatally exposed to BPA induce early vaginal opening (202). Neonatal SD rats were subcutaneously exposed to BPA, which caused multiple cystic follicles forming in the ovary (203). Similarly, an increase in cystic ovaries and pathologies of the oviduct and uterus were observed in mice (204). BPA-exposed ovaries showed a decreased antral & corpus luteum and increased atretic & cystic follicles (205). Maternal BPA exposure increases antral follicles' size, elevates the incidence of ovarian cysts, and changes ovarian follicular dynamics (206).

BPA exposure affects steroidogenesis in the ovary by reduced E2 production and decreased aromatase (CYP19A1) expression in human granulosa cells (207). Prenatal exposure to BPA caused alterations in fetal ovarian steroidogenesis by increasing Cyp19 and 5α-reductase expression and miRNA expression of folliculogenesis in sheep (208). Moreover, it was shown that patients with PCOS have decreased mRNA expression of aromatase (CYP19A1) compared to the non-PCOS group. Hyperandrogenism in PCOS is due to reduced aromatase activity (209). Bisphenol A exposure can also modulate hormone levels. Lower E2 levels and higher testosterone were observed in all the BPA-treated groups compared to the control in rat ovarian theca-interstitial and granulosa cells (210). On the contrary, LH and E2 significantly increase in the BPA-treated Wistar rats (211).

BPA is reported to modulate the HPO axis and elevate serum LH. Still, their low-level exposure *in utero* to develop a PCOS-like phenotype in the offspring must be reported. Despite the broader presence of direct hormone-regulated animal models in studying human PCOS, the pathogenesis and treatment still need to be determined. Thus, investigating the PCOS-like phenotype in animals without direct hormonal intervention is expected to determine the underlying mechanism for better understanding the pathogenesis of PCOS. Moreover, evidence from animal models may clarify the causal roles of EDC exposure in determining the PCOS risk factors.

## 7 Maternal phthalate exposure, endocrine effects on fetal growth and reproductive development

In the food processing, pharmaceutical, and medical industries, Phthalates are used as plasticizers in daily use products such as cosmetics, personal care, detergents, toys, household products, solvents, sealants, and paints. Men and women are differentially exposed to cosmetics. A recent study of participants (n=9218) investigated the exposure to EDCs based on occupation and gender differences in personal care product use. Higher urinary concentration of EDCs (BPA and mono-n-butyl phthalate, MnBP) was observed in women compared to men since the latter were exposed to phthalates-containing cosmetics more frequently than men (212). Women are suspected of exposure to various EDCs, including abundant phthalates in cosmetics. Women can carry and transmit this risk exposure to a new life. Numerous forms of phthalate exist in our environment, in which DEHP (Di 2-ethyl hexyl phthalate) is prominently present in food items. Samples originating from meats, fats, and dairy products may contain DHEP at concentrations (≥300 μg/kg) and significantly contribute to exposure in epidemiological studies. The molecular weight of DEHP is 390.56 g/mol. The first step in the metabolism of DEHP is the hydrolysis of DEHP to mono (2-ethylhexyl) phthalate (MEHP) catalyzed by unspecific lipases. Upon oral exposure, phthalates are metabolized quickly into their monoester metabolites. These phthalate metabolites have been detected in urine, maternal blood, amniotic fluid, and cord blood. In mice, MEHP is metabolized into a wide range of secondary metabolites (diacids and keto acids), excreted as glucuronide conjugates in human urine, and unconjugated metabolites in rat urine. A single oral dose clinical study found that about 71% of the DEHP delivery was excreted after 24 h, and 4% was excreted in the following 20h (213). European Food Safety Authority (EFSA) reported that a 50 μ*g*/kg/day dose could lead to testicular toxicity. *In utero*, DEHP exposure diminishes mineralocorticoid receptor expression in adult rat Leydig cells, affecting aldosterone-induced androgen formation and decreasing testosterone production. *In-utero* exposure to DEHP (100, 300, and 750 mg/kg/day) results in a 50% decrease in testosterone and aldosterone without changing corticosterone concentrations in male rats. DEHP was reported to enhance estrogenic activity at concentrations of 1.50 ppm *in vitro* and *in vivo*, suggesting its role in the transactivation of ER.

DEHP exposure decreased steroidogenic acute regulatory protein expression in pregnant mice and lowered fetal testicular 17α-hydroxylase and cytochrome P450 17A1 levels. Early trimester DEHP exposure is inversely associated with the anogenital distance of male newborns but not girls, indicating gender-specific effects on male genital development (214). In addition, the concentration of DEHP increases the chances of limb malformation in both litters and fetuses (215). The defects were specific to hind limbs and represented a spectrum of polydactyly phenotypes. In addition to the known effects of phthalate on fetal testicular functions, unexpected limb malformations and high rates of fetal death following exposure to 250 mg/kg DEHP was noted. However, further studies are needed to identify the mechanism responsible for DEHP-induced limb malformations and to determine the potential relevance of this finding concerning humans.

Due to their lipophilic nature, phthalates can act through nuclear membrane receptors and involves epigenetic modifications by altering the gene expression (216). Several animal studies and epidemiological data have been raised to define the risks of phthalates and their metabolites. Pregnant mice exposed to DEHP-induced fetal IUGR (217) lowered placental weight and reduced blood sinusoid area in the placental labyrinth layer in a stage-specific manner (218). Gestational DEHP exposure alters the expression of steroidogenic enzymes and circulatory steroid hormone levels in pregnant females (219). High doses of DEHP caused IUGR and induced fetal malformations (220). DEHP exposure also damaged DNA in the trophoblast (221) and altered the placental receptor expression (222).

Early exposure to phthalates can lead to metabolic, and reproductive diseases in later life. Gestational and lactation exposure to phthalates causes hyperglycemia, impaired glucose, and insulin tolerances, and altered insulin transduction pathways in adult male offspring (223). Gestational DEHP exposure results in impaired β-cell function by downregulating genes involved in their development and function by the increase in the global DNA methylation (224, 225). Recent data reported that maternal urinary phthalate metabolites are associated with DNA methylation profiling of the first-trimester placenta. The results showed 282 differentially methylated regions (DMRs) corresponding to 245 unique genes in the early human placenta for high compared to low total phthalate exposure. The phthalate metabolites in urine were related to changes in miRNA expression of the human placenta, where miR-142-3p, miR15a-5p, and miR-185 expression were associated between phthalate exposure and protein serine/threonine kinase activity (226). Phthalate exposure altered the first-trimester placental transcriptome and methylome connected with the placental growth factors (227). Maternal exposure to phthalates seems to promote the transgenerational inheritance of adult-onset disease risk factors in subsequent generations. Exposure of DHEP to F0 generation activated the PI3K/Akt/mTOR signaling in the germ cells of the adult mice testis in F1 and F2 generations (228). Prenatal DEHP exposure during gonads differentiation displayed symptoms similar to the human testicular dysgenesis syndrome in adult male offspring mice, which was causally associated with promoter silencing of genes involved in seminal vesicle secretory protein and antigens (229). Early and postnatal lactational DEHP exposure decreased testis weights and disturbed testis architecture by altering seminiferous tubule diameter and germinal epithelium height in adult offspring (230). Emerging epidemiological and clinical data on phthalates exposure as measured by their metabolites indicated pregnancy risks and skewed growth & development of the newborns. Maternal phthalates exposure was associated with fetal and neonatal growth parameters, including anogenital index, gestational age, birth length, birth weight, head circumference, and others (**Table 2**).

**Table 2:** Phthalates exposure, pregnancy risks and growth & development of the newborns: epidemiological and clinical data.

Subjects	Sample types	Parameters^#^	Key findings	Ref.
Pregnant women (n=463)	Urine and blood	Phthalate metabolites (12) and maternal hormones	-MnBP and ∑DEHP positively associated with TSH, while MEP and MnBP inversely associated with free thyroxine and total triiodothyronine	(231)
Mother-infant pairs (n=65)	Maternal blood and cord blood	Phthalate metabolites (10) and birth outcomes	-MBzP, MMP, MiBP, and ∑DEHP in maternal blood inversely correlated with the anogenital index (AGI) of male infants. -MOP, MMP, MiBP, MnBP, and MBzP wepositively correlated with the AGI of female infants. Cord blood levels of MnBP, mono-(2-ethyl-5-oxohexyl)-phthalate, MEHP, and ∑DEHP inversely associated with head circumference.	(149)
Pregnant women (n=165)	Urine	Phthalate metabolites (11) and birth outcomes	-Phthalate exposure inversely associated with shorter pregnancy and a decreased head circumference	(232)
Pregnant women (n=149)	Urine	Phthalate esters (9), PAHs and birth outcomes	-No effect of phthalate metabolite and PAHs on birth outcomes	(233)
Mother- new born pairs (n=72)	Urine, serum	Phthalate metabolites, birth outcomes and maternal hormones	-Positive correlation between neonatal anthropometric parameters (gestational age, birth length, birth weight, head circumference) and maternal concentration of phthalate metabolites	(234)
Pregnant women (n=121)	Urine	Phthalate metabolites (4) and anthropometry	-Association of maternal MBzP and MEHHP with the birth weight of female newborns. -MBP and MBzP negative associated with the head circumference in male and female newborns, respectively	(235)
Pregnant women (n=158)	Amniotic fluid	Phthalate metabolites (4) and anthropometry	-No association between phthalate metabolites and newborns anthropometric parameters.	(236)
Pregnant women (n=3474)	Urine	Phthalate metabolites (7) and birth weights	-MMP and MEP exposure during pregnancy associated with a decreased birth weight of infants	(237)
Pregnant women (n=3474)	Urine	Phthalate metabolites (7) and GDM	-MMP, MEP, MnBP, MBzP, and MEHHP exposure positively correlated with the fasting blood glucose in the third trimester, while MEHP and MEOHP exposure negatively correlated	(238)
Pregnant women (n=434)	Urine	Phthalate metabolites (19) and hormones	-Positive associations between phthalate metabolites, maternal estrogens, and testosterone	(239)
Pregnant women (n=299)	Urine	Phthalate metabolites (19) and gestational age-specific z-scores (GWGz)	-Sums of metabolites of ƩDEHP, ƩDiNCH, and ƩDEHTP had consistent inverse associations with GWGz	(240)
Pregnant women (n=677)	Urine and serum	Phthalate metabolites (19) and nine serum hormones	-Maternal testosterone positively associated with MHBP, and inversely associated with MEP. -Maternal CRH inversely associated with MCNP, MCPP, MECPP, MEHHP, and MEOHP.	(241)
Mother-infant pairs (n=553)	Urine and cord blood	Urinary phthalate metabolites and cord blood glucocorticoids	-MBzP in the first trimester associated with a higher cortisol/cortisone ratio. -MECPP and MEOHP measured in the third trimester were correlated with decreased cortisone.	(242)

^#^ The number of phthalate metabolites measured are indicated within bracket. Elaboration of abbreviated phthalate metabolites are followed as : Mono-2-ethyl-5-hydroxyhexyl phthalate (MEHHP); mono-2-ethyl-5-carboxypentyl phthalate (MECPP); mono-3-carboxypropyl phthalate (MCPP); mono carboxyisononyl phthalate (MCNP); mono hydroxybutyl phthalate (MHBP); di(isononyl) cyclohexane-1,2-dicarboxylate (ƩDiNCH); di(2-ethylhexyl) terephthalate (ƩDEHTP); Mono-isobutyl phthalate (MiBP); mono-oxo-iso-nonyl phthalate (MOiNP); mono-(2-ethyl-5-hydroxylhexyl) phthalate (MEOHP); mono-methyl phthalate (MMP); mono-ethyl phthalate (MEP); mono-n-butyl phthalate (MnBP); mono-n-octyl phthalate (MOP); mono-benzyl phthalate (MBzP); and the metabolite of di-2-ethylhexyl phthalate (DEHP), which were mono (2-ethylhexyl) phthalate (MEHP), mono-(2-ethyl-5-oxohexyl) phthalate (MEOHP), and mono-(2-ethyl-5-carboxypentyl) phthalate.

## 8 *In utero* phthalates exposure and testicular functions

**Figure 3:**
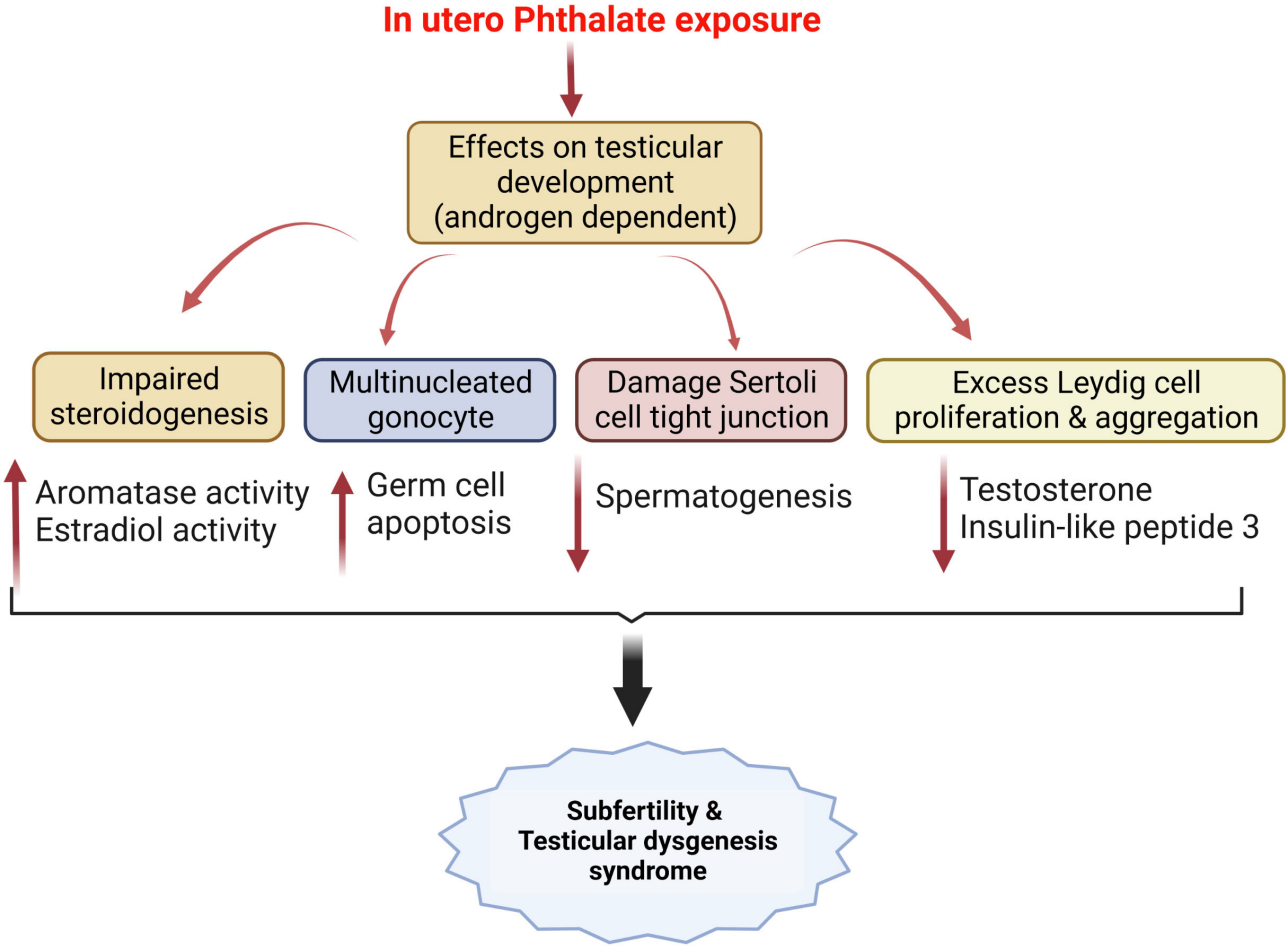
In utero phthalate exposure and its effects on testicular development modulates male fertility and results in testicular dysgenesis syndrome, as reported by several pre-clinical studies mentioned in the text.

Several in vivo studies show that phthalate exposure, irrespective of exposure stages can have long-term programming consequences in developing reproductive dysfunctions. Sprague Dawley dams were exposed to dibutyl phthalate (DBP) (2550 mg/kg) by oral gavage from gestational day 12 for 10 days, lowered the serum testosterone levels, caused fetal Leydig cell aggregation and lowered Lhcgr, Star, Insl3, and Nr5a1 levels in the male fetuses (243). Diisobutyl phthalate (DiBP) has structural and application properties similar to DBP and is used as a substitute for DBP. DiBP exposure to pregnant Wistar rats significantly reduced the male offspring’s anogenital distance and testicular production. The histology data showed Leydig cell hyperplasia, Sertoli cell vacuolization, and central location of gonocyte (244). In utero exposure to dipentyl phthalate (DPeP) from gD 14 to 21 decreased male offspring's serum testosterone and luteinizing hormone levels and increased the fetal Leydig cells by increasing proliferation (245). Gestational di-(2-ethylhexyl) phthalate (DEHP) exposure to SD rats significantly reduced serum testosterone levels, reduced fetal Leydig cell size, induced abnormal aggregation of these cells, and altered MDA levels in pup’s testis (246). Another synthetic phthalate plasticizer diisodecyl phthalate (DIDP), decreased serum testosterone, and induced abnormal aggregation of fetal Leydig cells. However, no effect on Leydig and Sertoli cell numbers was observed in the male pups (247). Diisopentyl phthalate (DiPeP) lowered mRNA levels of key steroidogenic proteins like Star, Cyp11a1, Cyp17a1, Ar, Esr1, Gper, and Insl3 in testis of prenatally exposed rats (248). In-utero exposure to diisononyl phthalate (DiNP) caused a reduction in fetal testosterone and Insl3 levels in male rats (249). Similar outcomes were observed with the exposure to unique phthalates such as bis (2-butoxyethyl) phthalate (BBOP) which contains oxygen atoms in the carbon backbone (250). In-vivo studies were conducted with mixtures of different phthalates to study their combined effect on testicular testosterone production. Co-administration of DBP and DEHP in pregnant SD rats reduced fetal testosterone levels in addition to reproductive malformations such as epididymal agenesis and reduced androgen-dependent organ weights (251). Similar findings were observed when co-administration of five phthalate esters i.e., benzylbutyl phthalate (BBP), di(n)butyl phthalate (DBP), diethylhexyl phthalate (DEHP), diisobutyl phthalate (DiBP) and dipentyl phthalate (DPP) in dams inhibited fetal testosterone production in the SD rats (252). In addition to testosterone reductions, phthalates impair sperm quality and induce inflammation of testicular Sertoli cells (253, 254). All these in vivo data indicate that phthalates lead to testicular dysgenesis syndrome irrespective of the type of the phthalate exposed (**Figure 3**).

## 9 Modulation of early development by epigenetic-DNA methylation: effects of endocrine disruptors

Epigenetic control of early development is susceptible to the factors associated with environmental and lifestyle practices since a set of genomic imprinters is programmed to execute their functions for early development. The window of fetoplacental development is plastic, capable of remodeling, and sensitive, during which changes in the maternal milieu by internal or external factors might significantly impact developmental programming. Epigenetic mechanisms involve multiple ways, including DNA methylation, histone modifications and microRNAs, and others. A dysregulated epigenetic-DNA methylation results in a change in fetal outcomes by regulating placental methylome and function (255). Maternal nutrition, stress, physical activity, hormones, and endocrine disruptors can modulate epigenetic control of fetal development during gestation. For example, maternal intake of alpha-linolenic acid during pregnancy and lactation causes changes in DNA methylation of the offspring’s liver (256), and maternal omega-3 deficiency increased 5-methylcytosine content in the mice’s placenta, indicating placenta’s sensitivity to epigenetic changes during development (257). Thus, a successful pregnancy requires controlled epigenetic modifications that lead to genome stability during developmental events, including organ formation from gamete production and fertilization to fetoplacental development and fetus outcome (258).

Unlike peptide hormones, steroid hormones are lipophilic that can cross cell membranes and signal through nuclear receptors, acting on transcription factors to turn on or off gene expression. Estrogen, a steroid hormone, binds to either membrane estrogen receptors (ER) or nuclear ERs. Endocrine disruptors like BPs are also known as synthetic estrogen. Activating a non-genomic pathway by binding estrogen to membrane ER causes activation of the PI3K-AKT signaling and downstream activation of gene transcription. Nuclear ER provides DNA-dependent regulation of gene expression mediated by binding of ER with coactivators or corepressors, which encode enzymes with histone acetyltransferase activity (259). RIZ1 is an ER coactivator, which represses transcription by methylation of lysine 9 of histone 3. Knockout of RIZ1 in female mice exhibit a decreased response to female sex hormones, reduced vaginal epithelial thickening in response to estrogen, and reduced litter sizes compared with their wild-type (260).

Although the role of epigenetics in successful pregnancy is well known, endocrine regulation of epigenetic processes is complex, and less data is available on the endocrine regulation of DNA methylation and histone modifications in response to endocrine disruptors like BPA. The sensitive window of early embryonic and placental development stages is more vulnerable as significant imprinting genes control these events. *In utero*, BPA exposure significantly altered the methylation levels of differentially methylated regions (DMR) imprinting control region and Igf2 DMR (261). Placental trophoblast cells respond to various exogenous agents by activating stress pathways, and epigenetic controls often regulate some of these responses. Data showed that alteration in DNA methylation profile in the first-trimester trophoblast genomic DNA correlated with CpG methylation of gene promoters associated with stress responses, including oxidative stress, inflammation, DNA damage, apoptosis, hypoxia, and the unfolded protein response (27).

Furthermore, the percentage of promoter methylation was lowered by BPA exposure compared with those of control cells. Normally, DNA methylation of gene promoters represses gene transcription to those that encode stress and toxicity pathways. However, stress response genes were de-repressed due to BPA exposure. In normal physiology, the trophoblast cortisol could protect the fetus by methylating genomic DNA to repress gene transcription of stress and toxicity mediators (262), which was unmethylated by the presence of BPA, indicating that activation of these pathways in response to BPA exposure by epigenetic modification. Recently we observed that gestational BPs exposure significantly raised plasma cortisol, body weight, and adiposity in 90d offspring (unpublished data). Thus, placental programming in DNA methylation due to BPA exposure could make the offspring susceptible to stress and anxiety behavior later in life.

BPA modulates gene expression through epigenetic modifications, such as DNA methylations, by either DNA hyper or hypo-methylation. The maternal exposure to BPA shifted the coat color of yellow Agouti mouse offspring toward yellow by decreasing CpG (cytosine-guanine dinucleotide) methylation in an intra-cisternal retrotransposon upstream of the Agouti gene (263). *In-utero* BPA exposure in pregnant CD-1 mice affects the expression of the homeobox (Hoxa10) gene by decreasing the promoter DNA methylation of CpG sites of the Hoxa10 gene in the reproductive tract of mice (193). In a recent study, the association between prenatal BPA exposure and DNA methylation in the placenta found that BPA causes differential hypo/hypermethylation of CpG sites in the BPA-exposed groups (264). Maternal BPA exposure caused promoter hypermethylation of three genes (CAPS2, TNFRSF25, and HKR1) in the cord blood (265). Perinatal BPA exposure results in abnormal DNA methylation in hepatic tissue, which precedes with the development of insulin resistance, indicating fetal reprogramming via epigenetic changes (266). Developmental exposure to BPA predisposed the offspring to progress steatohepatitis when fed a high-fat diet due to dysregulated gene expression in the liver (267). BPA exposure *in utero* induced preadipocyte differentiation by DNA hypermethylation of the CPT1A gene, indicating that BPA exposure induced obesity in the offspring by accumulating free fatty acids (268). Prenatal exposure to BPA altered the MEST promoter methylation, leading to increased risks of childhood obesity until about 6 years of age (269).

Further study confirmed that hypomethylation of the MEST promoter, a gene code for α/β hydrolase, is associated with obesity in mice exposed to BPA prenatally. Apart from being sex-specific, the tissue-specificity of prenatal exposure to BPA was also associated with the global methylation of long-interspersed nuclear element-1, a repetitive DNA element expressed in the liver (270). Maternal BPA exposure during gestation and lactation affected glucose homeostasis in the F2 offspring where hepatic glucokinase gene promoter was completely methylated in all CpG sites compared with unmethylated sites in controls of the exposed liver tissue, indicating BPA’s control on the metabolic fate of the offspring via epigenetic programming of metabolic gene expression (271). Furthermore, BPA-exposed mice exhibited preeclampsia-like features, including hypertension, disrupted angiogenesis biomarkers in circulation, and placenta’s involvement in the reprogramming of DNA methylation of WNT2/beta-catenin gene, indicating that exposure window of placental development is the most critical for the epigenetic targets, thereby, a key determinant for the progression of placental-disease preeclampsia (272).

Epigenetic changes are sensitive to environmental factors, including exposure to EDCs like phthalate. Phthalates can cross the placental barrier, and their metabolites have been detected in the placenta. During pregnancy, the placenta produces and releases diverse miRNAs into the maternal circulation, which can be modulated due to the impact of phthalate exposure. Studies have indicated that phthalates and their metabolites can alter miRNA profiles in placental tissue, suggesting a potential link between phthalate exposure, miRNA dysregulation, and adverse pregnancy outcomes. Urinary levels of 13 phthalates in pregnant women with uncomplicated term dichorionic, diamniotic twin pregnancies and the miRNA profile of placental EVs (EV-miRNAs) circulating in maternal blood were measured. The expression of miR-518e in the maternal blood was highest among women with high mono-benzyl phthalate Field urinary levels (273). A study on 179 women found associations between first-trimester phthalate levels and miRNA expression in the placenta. miR-142-3p, miR15a-5p, and miR-185 expressions were found to be associated with urine phthalate levels (226). Mono-(2-ethylhexyl) phthalate (MEHP) induces oxidative stress-responsive miR-17-5p, miR-155-5p, and miR-126-3p in HTR8/SVneo in a dose- and time-dependent manner by altering the expression of genes involved in oxidative stress (274). A clinical study conducted on 202 pregnant women between 22 and 29 gestational weeks measured 11 phthalate metabolites in the spot urine samples and global DNA methylation in the placental tissue of the fetal side at delivery. Mono-benzyl phthalate concentration was inversely associated with the placental methylation of Alu repeats.

Moreover, all phthalate biomarkers except for mono-carboxy-iso-octyl phthalate and mono (2-ethyl-5-hydroxyhexyl) (MEHHP) phthalate were associated with at least one differentially methylated region (275). A case-control study examined the associations of prenatal phthalate exposure, infant growth, and global DNA methylation in human placenta 119 subjects [55 fetal growth restriction (FGR) cases and 64 normal controls]. Concentrations of mono (2-ethyl-5-hydroxyhexyl) phthalate, mono (2-ethyl-5-oxohexyl) phthalate (MEOHP), and sum DEHP (molar sum of MEHP, MEHHP, and MEOHP) were significantly higher in FGR cases than those in normal controls. Placental LINE-1 methylation was positively associated with fetal birth weight and negatively associated with urinary phthalate metabolites concentrations (MEHHP and mean DEHP) (276). Another study conducted in 181 mother-newborn pairs (80 fetal growth restriction newborns, 101 normal newborns) measured third-trimester urinary phthalate metabolite concentrations and placental DNA methylation levels of IGF2 and found that urinary concentrations of MEHHP and MEOHP were inversely associated significantly with placental IGF2 DNA methylation (277). A recent study measured the effect of maternal phthalate exposure on the human placental DNA methylome, transcriptome and identified 39 genes with significantly altered methylation and gene expression in the high phthalate exposure group, with most of these relationships were inversely correlated (29 out of 39) (227).

Moreover, placental imprinting was assessed at birth in placental samples in 179 subjects and found a significant decrease in H19 methylation, which was associated with high levels of the sum of phthalate metabolites and metabolites of low molecular weight (LMW) phthalates. Σ phthalate and LMW phthalate concentrations were inversely associated with IGF2 differentially methylated regions methylation values (278). Placental exposure to phthalates may result in aberrant miRNA expression. Pregnant SD rats exposed to DEHP promoted the placental miR-155-5p expression, the cAMP/PKA inactivation, lipid metabolism and altered placental histopathology. Moreover, the knockdown of miR-155-5p abrogated DEHP-induced proliferative, migrative, and invasive inhibition in HTR8/SVneo cells (279). Phthalates (MEHP) have been reported to induce apoptosis by inducing miR-16, which alters the BCL-2/BAX ratio in the first trimer placental trophoblast HTR-8/SVneo cells (280).

Available data indicate that gestational phthalate exposure could modulate the placental gene expression related to its function or altered response to the fetus due to aberrant expression of miRNAs in the placenta.

## 10 Conclusion

EDCs exposure can significantly affect fetoplacental growth and pregnancy outcomes via different mechanisms. Several EDCs are estrogen-mimicking steroids, and those can quickly diffuse into the nucleus across the cell membrane and modulate the transcription of genes. These endocrine disruptors can mediate their effects by interfering endocrine function of the hypothalamic-pituitary glands, changing the secretion of gonadotropin-releasing hormone in the hypothalamus, promoting the proliferation of the pituitary, premature puberty, and resulting in infertility risks in both men and women. The epigenetic fingerprint of steroidogenesis and organogenesis in the uterus, follicles, implant, and placenta could risk the gonadal programming of the offspring. The most abundant EDCs include BPs, which report obesogenic effects, disrupting regular metabolic activity, making the body prone to overweight, and obesity. Being overweight and obese state impedes a successful pregnancy. In addition, prenatal BPs exposure may influence postnatal fetal HPA responsiveness due to alterations in cortisol levels and HSD11β activities.

Several epidemiological studies pointed out maternal exposure to BPs and Phthalates is associated with adverse outcomes on fetal growth and development, its life-long impact in the endocrine-linked growth process, and risks of reproductive organ deformities. However, data correlating of these EDC exposure with the early stage of puberty and sexual maturity and its follow-up with pre-pregnancy endocrine regulation is required for renewing better strategies in preventing EDCs-exposure induced harmful effects on pregnancy outcomes.

## Author contributions

SB: writing, revision, and approval of the final version. SV: writing. AD: revision and approval of the last version. All authors have approved the final version for submission.
